# Infectious complications of bispecific antibody therapy in patients with multiple myeloma

**DOI:** 10.1038/s41408-023-00808-8

**Published:** 2023-03-10

**Authors:** Beatrice Z. Sim, Anthony Longhitano, Jeremy Er, Simon J. Harrison, Monica A. Slavin, Benjamin W. Teh

**Affiliations:** 1 Department of Infectious Diseases, Peter MacCallum Cancer Centre, Parkville, VIC Australia; 2 National Centre for Infections in Cancer, Parkville, VIC Australia; 3 Sir Peter MacCallum Department of Oncology, University of Melbourne, Parkville, VIC Australia; 4 Department of Clinical Haematology, Peter MacCallum Cancer Centre and Royal Melbourne Hospital, Parkville, VIC Australia; 5 Department of Medical Biology, The Walter and Eliza Hall Institute of Medical Research, Parkville, VIC Australia

**Keywords:** Infectious diseases, Myeloma

## Introduction

Infection has a significant impact on patients with multiple myeloma (MM) [1]. Bispecific antibody (BsAb) therapy with multiple targets (BCMA, GPRC5D and FCRH5) is increasingly used with good disease response rates [2]. Trial data on infections has been limited, with incidences of all-grade infections reported at 13–76% and grade ≥3 at 3–45% [3, 4]. However, the diagnostic criteria used and infection episodes were not well characterised. Therefore this study was conducted to describe the epidemiology and outcomes of infections in BsAb treated MM patients to better guide antimicrobial prophylaxis and treatment in this emerging cohort.

## Methods

All MM patients who received BsAb therapy on a range of clinical trials between 1st January 2018 and 30th May 2022 at Peter MacCallum Cancer Centre were included in this retrospective review of patients’ electronic medical record. All demographic, disease and infection-related data including outcomes were collected utilising a standardised case report form. Patients were followed up for 6 months post last BsAb dose, until next treatment line or death, whichever occurred later. At our institution, valaciclovir 500 mg daily and trimethoprim/sulfamethoxazole daily is routinely used for herpes simplex, zoster and Pneumocystis prophylaxis respectively while antibacterial prophylaxis is not routinely used. Antifungal prophylaxis is guided by an individualised risk-based approach (e.g lines of prior therapy, previous infection).

Complications of BsAb therapy such as cytokine release syndrome (CRS) and immune effector cell-associated neurotoxicity syndrome (ICANS) were defined according to published criteria [5]. Infection episodes were categorised as microbiologically-defined (MDI), clinically-defined (CDI) or fever of unknown focus (FUF) and severity graded according to published criteria [6, 7]. MDI was defined as laboratory-isolated pathogen with a compatible clinical syndrome, CDI was defined by compatible clinical syndrome/site without an isolated pathogen, and FUF was defined as presence of fever and the absence of compatible microbiology or focal symptoms.

Descriptive summary statistics were performed. Univariate and multivariate logistic regression were performed to identify factors associated with MDI. Statistical analysis was conducted on Rstudio, 2021.9.0.351 (USA) with *p* < 0.05 considered statistically significant.

## Results

We identified 39 MM patients who received BsAb therapy at our centre with median follow-up of 152 (IQR 51–277) days. The median age was 62 years (IQR 56–70), 24 (62%) were male and had received a median of 6 (IQR 4–7) prior lines of therapy. The majority of patients (38, 97%) had prior haematopoeitic stem cell transplant.

Patients completed a median of 5 (IQR 2–11) cycles of BsAb therapy and CRS occurred in 28 (72%) and ICANS in 2 (5%) patients. The median duration of neutropenia and lymphopenia <1.0 × 10^9^/L was 1 (IQR 0–3) and 22 (13–61) days respectively. Hypogammaglobulinemia was documented in 34 (87%) patients, with immunoglobulin replacement in 18 (53%). Further details are summarised in Table 1.

**Table 1:** Baseline characteristics of the 39 MM patients on BsAb therapy.

Characteristic	Detail (*n* = 39)
Demographics	
Age (years; median, IQR)	62 (56–70)
Sex (*n*, %)	M: 24 (62) F: 15 (38)
ECOG (*n*, %)	0: 10 (26) 1: 29 (74)
CCI^1^ (median, IQR)	5 (4–7)
Myeloma details (*n*, %)	
Multiple myeloma type	IgG: 26 (67) IgA: 9 (23) Light chain: 4 (10)
ISS^2^	1: 11 (28) 2: 17 (44) 3: 11 (28)
Treatment	
Number of previous treatment lines (median, IQR)	6 (4–7)
Total number of BsAb cycles (median, IQR)	5 (2–11)
BsAb targets^2^	BCMA: 12 (31)^3^ Non-BCMA: 27 (69)
Duration (days; median, IQR)	85 (39–236)
Adverse events	
Cytokine release syndrome (*n*, %)	28 (72)
Immune effector cell-associated neurotoxicity syndrome (*n*, %)	2 (5)
Duration of neutropenia <1 × 10^9^/L (days; median, IQR)	1 (0–3)
Duration of lymphopenia <1 × 10^9^/L (days; median, IQR)	22 (13–61)

^1^Charlson Comorbidity Index.

^2^International Staging System Score.

^2^BsAb: bi-specific antibody.

^3^BCMA: B-cell maturation antigen.

Of the 39 patients, 35 (90%) had at least 1 episode of infection (MDI, CDI, FUF). A total of 15 (38%) had an MDI and 16 (41%) had at least one grade 3 or higher infection episode (Table 2).

**Table 2:** Characteristics and outcomes of infection episodes.

Characteristic	Detail (*n* = 111)
Treatment course	
BsAb cycles at time of infection (median, IQR)	2 (1–7)
Cumulative prednisone-equivalent dose, 30 days (mg; median, IQR)	266.7 (106.7–266.7)
Lowest neutrophil level, 14 days prior (x10^9^/L; median, IQR)	1.8 (1.1–2.3)
Lowest lymphocyte count, 14 days prior (x10^9^/L; median, IQR)	0.3 (0.1–0.7)
Prophylaxis (*n*, %)	
Antiviral	Valaciclovir: 104 (94) Valganciclovir: 3 (3) Famciclovir: 3 (3) None: 1 (1)
Antifungal	Fluconazole: 9 (8) Mould-active antifungal: 29 (26) Posaconazole: 24 (22) Voriconazole: 5 (5) None: 73 (66)
* P. jirovecii* prophylaxis	Trimethoprim/Sulfamethoxazole: 104 (94) Dapsone: 5 (5) None: 2 (2)
Infection details	
Number of patients (*n*, %)	35 (90)
Grade of infection	1: 37 (33) 2: 50 (45) 3: 22 (20) 4: 0 (0) 5: 2 (2)
Infection category^!^	MDI: 33 (30) CDI: 43 (39) FUF: 35 (32)
Site of infection	Respiratory: 46 (41) Urinary: 4 (4) Bloodstream: 6 (5) Skin and soft tissue: 4 (4) Gastrointestinal tract: 8 (7) Ear nose and throat: 6 (5) Genital: 2 (2) CRS: 31 (28) Unknown: 4 (4)
Pathogen details (*n*, %)	
Pathogens isolated^a^	Bacterial: 15 (39) Gram Negative: 12 (32)^1^ Gram Positive: 3 (8)^2^ Viral: 22 (58) Respiratory viruses: 15 (39)^3^ Reactivated viruses: 7 (18)^4^ AFB: 1 (3)^5^
Treatment and outcomes	
Total antibiotic duration (days; median, IQR) IV PO	5 (3–9) 3 (3–4) 5 (5–10)
Hospital admission (*n*, %)	63 (57)
Outcome at 30 days (*n*, %)	Survival: 108 (97) Death: 3 (3)^6^

^!^*MDI* microbiologically diagnosed infection, *CDI* clinically diagnosed infection, *FUF* fever of unknown focus.

^a^There were 2 pathogens isolated in 5 infective episodes: adenovirus & EBV, *Haemophilus influenza* & rhinovirus, influenza & adenovirus, *S. aureus* & *E. coli, Bordetella parapertussis* & rhinovirus/enterovirus.

^1^*Salmonella sp.* (*n* = 3), Bordetella parapertussis (*n* = 1), *Escherichia coli* (*n* = 3), *Pseudomonas aeruginosa* (*n* = 1), *Haemophilus influenzae* (*n* = 2), *Klebsiella pneumoniae* (*n* = 1), *Proteus mirabilis* (*n* = 1).

^2^*Clostridium difficile* (*n* = 1), *Enterococcus faecalis* (*n* = 1), *Staphylococcus aureus* (*n* = 1).

^3^Rhinovirus/enterovirus (*n* = 8), influenza (*n* = 1), adenovirus (*n* = 4), COVID-19 (*n* = 2).

^4^BK virus (*n* = 1), cytomegalovirus (*n* = 4), herpes simplex virus 2 (*n* = 1), Epstein Barr virus (*n* = 1).

^5^*Mycobacterium avium* complex (*n* = 1).

^6^Deaths attributed to preceding infection within 30 days.

A total of 111 infection episodes occurred during the study period at a median of 2 (IQR 1–7) BsAb cycles. Median (IQR) nadir neutrophil and lymphocyte counts in the preceding fortnight were 1.8 (1.1–2.3) × 10^9^/L and 0.3 (0.1–0.7)) × 10^9^/L respectively. Prophylactic valaciclovir had been administered in 104 (94%), trimethoprim/sulfamethoxazole in 104 (94%) and antifungal prophylaxis (most commonly mould-active) in 38 (34%) infection episodes. Median cumulative prednisone-equivalent dose received by patients in the 30 days prior to infection was 266.7 mg (IQR 106.7–266.7).

Of the 111 infective episodes, 33 (30%) were MDI, 43 (39%) were CDI, and 35 (32%) were FUF (Table 2). The most common site of infection was the respiratory system (46, 41%) followed by gastrointestinal (8, 7%). Of the MDI, 22 (58%) viruses were isolated, 15 (39%) bacteria, and 1 (3%) acid-fast bacilli. Two organisms were isolated in 5 infective episodes. The most common viral infection was rhinovirus/enterovirus (8, 36%), followed by cytomegalovirus (4, 18%) and adenovirus (4, 18%). Majority of bacterial infections were associated with the gastrointestinal tract (*Salmonella* sp., *Escherichia coli*, *Pseudomonas aeruginosa, Clostridium difficile*, *Enterococcus faecalis*). There were no episodes of invasive fungal disease. MDI episodes occurred a median of 79 (IQR 29–289) days after BsAb commencement. Majority of FUF were likely CRS (31, 89%).

Sixty three (57%) infective episodes, in 30 patients, resulted in hospital admission with a median length of stay of 4 (3–7) days. Intensive care admission was required in 4 (4%) episodes and all-cause mortality was 3%. Patients required a median (IQR) of 5 (3–9) days of intravenous or oral antibiotics.

Baseline clinical variables age, functional status, international staging system stage, prior lines of therapy, number of BsAb cycles, CRS, and its treatment were not associated with increased risk for MDI on univariate analysis. Therefore multivariate logistic regression was not performed.

## Discussion

Infection remains a clinically significant burden in heavily-treated MM patients undergoing BsAb therapy. Rates of infection associated with BsAb therapy have been variably reported in clinical trials. Most commonly used T cell/BCMA targeting BsAb trials have reported all-grade infection at 41–76% and grade ≥3 at 5–24% [3, 8] while the rates are 13–37% and 3–4% respectively in T cell/GPRC5D trials [3]. A trial of T cell/FCRH5 BsAb reported grade ≥3 infection of 19% and limited data is available for NK targeting BsAb [3, 9]. Real world data on rates of hypogammaglobulinaemia, impact of immunoglobulin replacement, and associated rates of infection are starting to emerge, however more evidence is necessary [10, 11].

In this study of heterogenous BsAb platforms, incidence of any infection was comparable to current generation of therapies for relapsed disease including monoclonal antibody therapy with 90% of patients with median 6 lines of prior therapy experiencing at least 1 episode of infection [12]. Severe infection rate was similar also at around 40% [12]. Infections involving the respiratory tract were most common, in keeping with previous literature [13, 14].

Viral infections were particularly prominent in this study, making up 58% of MDI. These were most commonly respiratory infections (15, 39%), particularly rhinovirus/enterovirus (8, 36%), followed by adenovirus, SARS-CoV-2 and influenza. Therefore surveillance and general prevention measures for respiratory viruses should be enhanced for these patients, and development of new treatment and prevention strategies should be prioritised. Although reactivation of viruses only made up 7% of MDI, infections were predominantly with viruses such as cytomegalovirus that are not preventable with standard prophylaxis highlighting the need for vigilance, early testing and treatment.

Despite a median of 6 prior lines of therapy, there were no episodes of invasive fungal disease detected. In contrast, the rate of IFD in MM patients treated with other new generation therapies was 3.4% [15]. However, reflective of a heavily-treated MM cohort and the uncertainty around the risk for fungal infection, a diverse a range of anti-fungal prophylaxis strategies was observed in this study. The optimal approach require further study.

While all-cause mortality and intensive care admission rates were low (3 and 4% respectively), infectious episodes resulted in a high rate of admission (57%) and prolonged antibiotic use, highlighting the need for improved prevention strategies to minimise the burden of infection, especially for therapies that are continually utilised until disease progression.

This study is limited by its relatively small cohort size and its retrospective nature. However, it is the first report to fully characterise infections in MM patients treated with BsAb.

Infection remains a significant burden for MM patients treated with BsAb with severe infection rates of 41% and viral infections predominate. Further studies are required in order to optimise prophylactic and preventative strategies.

## Data Availability

The datasets generated during and/or analysed during the current study are available from the corresponding author on reasonable request.
